# Medical decision-making among Hispanics and non-Hispanic Whites with chronic back and knee pain: A qualitative study

**DOI:** 10.1186/1471-2474-12-78

**Published:** 2011-04-21

**Authors:** Jeffrey N Katz, Nancy Lyons, Lisa S Wolff, Jodie Silverman, Parastu Emrani, Holly L Holt, Kelly L Corbett, Agustin Escalante, Elena Losina

**Affiliations:** 1 Orthopaedic and Arthritis Center for Outcomes Research, Brigham and Women's Hospital, 75 Francis St. Boston, MA 02115 USA; 2 Department of Orthopaedic Surgery, Brigham and Women's Hospital, 75 Francis St. Boston, MA 02115 USA; 3 Division of Rheumatology, Immunology and Allergy, Brigham and Women's Hospital, 75 Francis St. Boston, MA 02115 USA; 4 Harvard Medical School, Longwood Ave, Boston, MA 02115, USA; 5 Department of Epidemiology, Harvard School of Public Health, 677 Huntington Ave, Boston, MA 02115, USA; 6 Health Resources in Action, 95 Berkeley Street, Boston, MA 02116, USA; 7 Division of Rheumatology, University of Texas Health Science Center, 7703 Floyd Curl Dr., San Antonio, TX 78229, USA; 8 Department of Biostatistics, Boston University School of Public Health, 715 Albany St. Boston, MA 02118, USA

## Abstract

**Background:**

Musculoskeletal disorders affect all racial and ethnic groups, including Hispanics. Because these disorders are not life-threatening, decision-making is generally preference-based. Little is known about whether Hispanics in the U.S. differ from non-Hispanic Whites with respect to key decision making preferences.

**Methods:**

We assembled six focus groups of Hispanic and non-Hispanic White patients with chronic back or knee pain at an urban medical center to discuss management of their conditions and the roles they preferred in medical decision-making. Hispanic groups were further stratified by socioeconomic status, using neighborhood characteristics as proxy measures. Discussions were led by a moderator, taped, transcribed and analyzed using a grounded theory approach.

**Results:**

The analysis revealed ethnic differences in several areas pertinent to medical decision-making. Specifically, Hispanic participants were more likely to permit their physician to take the predominant role in making health decisions. Also, Hispanics of lower socioeconomic status generally preferred to use non-internet sources of health information to make medical decisions and to rely on advice obtained by word of mouth. Hispanics emphasized the role of faith and religion in coping with musculoskeletal disability. The analysis also revealed broad areas of concordance across ethnic strata including the primary role that pain and achieving pain relief play in patients' experiences and decisions.

**Conclusions:**

These findings suggest differences between Hispanics and non-Hispanic Whites in preferred information sources and decision-making roles. These findings are hypothesis-generating. If confirmed in further research, they may inform the development of interventions to enhance preference-based decision-making among Hispanics.

## Background

Musculoskeletal problems are prevalent, disabling and resource-intensive [1]. Utilization of health care services for these disorders differs across racial and ethnic groups. For example, Hispanic utilization of total hip and knee replacement in the United States is as low as half that of non-Hispanic Whites [2-5]. As one in eight U.S. residents was either born in a Spanish-speaking country or is related to someone who was [6-8], understanding these differences merits high priority.

The lower rates of total joint replacement among Hispanics do not appear to be attributable to ethnic differences in prevalence of arthritis, health insurance status, income or geographic location [2,3]. Possible explanations for the disparity include differences in the treatments that physicians recommend to Hispanic patients, and differences between Hispanic and non-Hispanic patients in their preferences for treatment. Since musculoskeletal disorders are not life-threatening, treatment decisions are sensitive to patient preferences. Shared decision-making (SDM) - a process that enables patients to make fully informed, preference-consonant decisions - has been proposed as an ideal process for making therapeutic decisions in such preference-sensitive situations [9-14]. These concepts of shared decision-making are embodied in the Ottawa Framework for Decision making, a well established paradigm used to evaluate the decision making process [15,16].

The shared decision-making process calls for patients to be offered descriptions of available treatment options and informed of the favorable and adverse outcomes associated with each one. The clinician also helps patients to assess their preferences for these outcomes. This process may be especially challenging for many Hispanics, who do not share the predominant culture and language and often have less formal education and lower health literacy than the general population [6]. Prior literature suggests that Hispanics may differ from non-Hispanic whites in the type of health information they seek, the extent of decision-making authority they wish to exercise and the role of religion and faith in decision-making [17-19].

These considerations led us to pose a series of questions: Do Hispanics obtain knowledge about treatment options in a fashion that is similar to Whites? Do Hispanics prefer to defer important health care decisions to their physicians or to the advice of family and friends? Given the substantial socioeconomic differences that exist among Hispanics in the U.S., we also ask whether the answers to these research questions differ according to socioeconomic indicators. Because our research questions have been largely unexamined in the arthritis and pain literatures, we chose to use qualitative methods to develop hypotheses that could then be tested quantitatively in subsequent research.

## Methods

### Design overview

We conducted six focus groups for Hispanic and non-Hispanic White subjects with chronic back or knee pain. The Hispanic and non-Hispanic White subjects were matched with respect to the socioeconomic features of their zip codes of residence. The groups were led by a professional moderator who used a semi-structured moderator's guide. We transcribed the focus group discussions and analyzed the transcripts using a grounded theory approach [17,20-22].

### Patient sample

#### Eligibility criteria

To be eligible for this study, subjects had to be older than eighteen and had to be seen at least twice in 2006 at Brigham and Women's Hospital for back or knee pain. We excluded subjects that lived outside of Route 95, a beltway around the Boston metropolitan area with radius of approximately ten to fifteen miles. This exclusion enhanced comparability of Hispanics and non-Hispanic Whites, as eligible Hispanic subjects rarely lived far outside of the city.

Hispanic ethnicity was confirmed by self-report using the 2000 U.S. Census criterion. We first asked the subjects' ethnicity (Hispanic or non-Hispanic), then asked the subjects' race. We excluded subjects who identified themselves as African American, Asian, Pacific Islander and Native American so that the two groups would be composed of Hispanics and non-Hispanic Whites.

#### Socioeconomic stratification

We obtained zip codes on all eligible subjects from the administrative database. The zip codes were grouped according to a variable we termed "vulnerability index," which was constructed as follows. First, we used 2000 U.S. Census data to characterize each zip code with respect to four variables: the proportion of residents in the subjects' zip code who had attained less than high school education; the proportion of residents in the zip code whose income was below the poverty line; the proportion who were foreign-born; and the proportion of racial and ethnic minorities. For each of these variables, we created a threshold value on distributional grounds. These included: >25% with income less than poverty; >28% with education less than high school; >26% foreign born and >64% non-white. If a zip code exceeded the threshold we regarded residents of that zip code as "vulnerable" with respect to that factor. We then stratified patients according to whether their zip code had one or fewer of these vulnerability factors ('less vulnerable') or two or more vulnerability factors ('most vulnerable').

### Recruitment procedures

#### Hispanic subjects

We identified our sample using the hospital's Research Patient Data Repository, a database that integrates information from diverse hospital electronic databases for research purposes. We performed a search within the Repository database for patients seen in outpatient practices for back or knee pain. Hispanic ethnicity was provisionally identified using the race/ethnicity indicator in the database. (We subsequently confirmed ethnicity in direct conversations with patients, as described above.)

We stratified Hispanic patients first into those with back versus knee pain. We further stratified according to whether their zip code had 0-1 vulnerability factors versus 2 or more. We attempted to recruit sufficient patients to conduct four focus groups among Hispanic subjects: two among Hispanics with two or more vulnerability factors (one of patients with knee pain, one with back pain) and two among Hispanics with two or more vulnerability factors. We envisioned, correctly, that we would reach saturation with four groups: that is, four groups were sufficient to elicit a wide range of responses, with few new pieces of information encountered in the latter groups. Ultimately, we did not receive enough responses from Hispanics with 0-1 vulnerability factors and knee pain. Therefore, we created a combined knee and back pain group of Hispanics with vulnerability score of 0-1. We sent a letter to patients (written in both Spanish and English to Hispanic patients) inviting them to participate. Patients could refuse by returning an opt-out letter. We called those who did not refuse to determine whether they were interested in participating. A research associate fluent in Spanish made the calls.

#### Non-Hispanic White Subjects

Each time a Hispanic subject agreed to participate, we selected three potential non-Hispanic White patients matched on clinical condition (back pain vs. knee pain) and the zip code-based vulnerability index. We invited these patients to participate. Our goal was to recruit one non-Hispanic White focus group with knee pain and one with back pain. The vulnerability groupings were admixed among non-Hispanic Whites, as resources precluded our including both high and low vulnerability White groups.

### Focus group protocol

Experienced moderators led the focus groups. A native Spanish speaker moderated groups comprised of Hispanic subjects. The moderators adhered to standard focus group procedures. These included maintaining neutrality; ensuring that concepts were understood before they were discussed; maintaining an environment in which all participants felt comfortable sharing their thoughts and experiences; eliciting comments from all participants; and ensuring confidentiality.

The focus groups were held in the evening. Participants completed a brief questionnaire upon arrival, including their level of educational attainment. Dinner was provided, along with travel expenses and a $40 honorarium. None of the subjects' health care providers attended the focus groups. The Spanish language focus group transcripts were transcribed in English by a bilingual speaker with substantial experience in Spanish-to-English transcription.

### Moderator's guide

We developed a semi-structured moderator's guide to ensure that all focus groups covered the same material and that the conversations covered the same key domains. The broad areas covered by the moderator's guide included knowledge of treatment options; the treatment decision-making process; discussions between patients and physicians about treatments; preferences for information resources and for roles in the decision process; and attitudes about strong or invasive treatment.

### Analysis

The analysis proceeded in two stages. In the first stage, one co-investigator (LW) read the transcripts and prepared a report identifying key themes. The report also emphasized differences among the Hispanic and non-Hispanic participants with respect to these themes. The research team then performed a second analysis that used a content analysis approach to identify themes and provide a semi-quantitative assessment of the differences across focus groups.

Accordingly, two investigators (KC, HLH) who did not attend the focus groups and were not involved in the development of the project proposal or moderator's guide analyzed the transcripts. These investigators read the transcripts to identify content areas without specific hypotheses guiding the identification of these areas. In this way, theory could be developed based upon observation of the data and not on prior assumptions [23,24]. After statements made by the subjects were allocated to content areas, the investigators reviewed the statements and identified specific research themes suggested by the statements. Each theme was framed directionally so that statements in the transcripts could be coded as either supporting the theme, refuting it, or neither supporting nor refuting the theme. The investigators coded all transcripts in this fashion. Two researchers (KC, HLH) coded two of these transcripts independently as described above. They then compared the results of their coding of these transcripts and discussed and resolved areas in which they disagreed. In this fashion, the two investigators developed a common approach. They then coded the remainder of the transcripts individually.

We tallied the number of statements supporting the theme, the number refuting and the number neutral with respect to the theme. We compared these data across the six focus groups. We used these data to produce summary tables and we supported the trends noted in the tables with representative quotations from the transcripts. We recognize that these tallies do not account for the possibility that a singe participant or two can contribute a large number of statements. We use the tallies as a rough gauge (rather than a precise estimate) of the comparative importance of themes arising in the focus groups.

The study was approved by the Brigham and Women's Institutional Review Board.

## Results

### Recruitment

Six focus groups were assembled, as shown in Table 1. Ninety percent of all participants were female. The median age of non-Hispanic White patients (back and knee groups) was 60 (range 47- 80). Among Hispanics, the median age of subjects with knee pain was 60 (range 40 - 83) and median age of subjects with back pain was 49 (range 32 - 65). Eighty-six percent of non-Hispanic Whites had attended college as compared with 24% of Hispanics.

**Table 1:** Number of subjects in each focus group (N = 39)

	Non Hispanic Whites	Hispanics	
	**Mixed SES**	**Low vulnerability**	**High Vulnerability**

Knee	6	6*	3

Back	8	9	7

*mixed knee and back

### Themes

The coding produced 36 distinct themes, though many of them were mentioned only rarely. We concentrated the analysis on the 21 themes that were noted more than nine times across the six groups. These are shown in Table 2, which documents the number times each theme was mentioned in each of the six focus groups.

**Table 2:** Number of mentions of key research themes, stratified by ethnicity, vulnerability group, and clinical condition

Ethnicity	Caucasian		Hispanic			
**Vulnerability**	**Mixed**		**High**		**Low**	

**Clinical**	**Back**	**Knee**	**Back**	**Knee**	**Back**	**Knee**

*Pain and Function:*						

Pain limits function	17	10	13	12	7	15

Pain 1° factor prompting care seeking	11	13	8	11	16	15

Treatments relieved pain	7	4	7	2	14	7

Treatments did not relieve pain	11	5	4	7	5	12

*Decision making:*						

Fear of bad treatment outcome inhibited patient from seeking care	2	5	12	4	9	3

Patient was aware of pros and cons of treatment	13	9	13	8	22	8

Prior experience with treatment made patient less likely to choose treatment	2	0	1	0	3	6

Prior experience with treatment made patient more likely to choose treatment	4	4	1	2	3	2

Primary decision maker is NOT the patient	3	2	1	1	12	6

Primary decision maker is the patient	15	5	3	9	34	13

*Patient-physician relationship*						

Patient distrusts the physician	3	3	4	0	2	0

Patient trusts the physician	12	6	5	8	15	7

Patient dissatisfied with physician's advice	21	3	6	3	6	4

Patient satisfied with physician's advice	7	5	2	4	9	4

Patients dissatisfied with physician's manner and rapport	5	6	3	0	6	5

Patients satisfied with physician's manner and rapport	9	9	1	0	7	2

Patients dissatisfied with medical practice's service	9	1	3	1	9	4

Patients satisfied with practice's service	0	3	0	4	6	1

*Coping resources*						

A positive attitude helps patients cope with pain before and after treatment	0	0	3	1	8	0

Faith and religion helps patients cope with pain before and after treatment	0	0	5	2	8	1

Family members will care for a patient recovering from surgery	0	1	1	0	3	1

#### Role of Pain in Function and Decisions regarding Care-Seeking

Participants across all six focus groups made numerous statements indicating that pain was the primary reason they sought care for their musculoskeletal problem. They expressed frustration with pain and with treatments that did not manage pain adequately.

- "I feel more, more, more nervous, frustrated, more desperate because of the pain."-Hispanic participant

- "I've told the doctor, that when the pain is too strong, I drink more pills than what they prescribe. When I can't bear the pain anymore, I drink more [medication], because it's just unbearable."-Hispanic participant

- "You can't walk any distances [with the pain], you can't stand in line at Wal-Mart...it overtakes your life like that. And I was to the point where I just, I felt old...."-Non-Hispanic White participant

#### Decision-making

Numerous statements in each of the six focus groups indicated that the participants were aware of the pros and cons of various treatments. However, differences emerged in the role of prior experiences on decisions to seek care or choose treatments. As compared with Non-Hispanic Whites, Hispanic subjects made more statements indicating that fear of negative treatment outcomes inhibited them from seeking care. Similarly, Hispanics were more likely to comment that adverse prior treatment experiences of friends and family made them *less inclined *to choose a particular treatment.

"I worked with a person who had arthritis and she was going to get cortisone and the last time she went to get it at the hospital, she didn't return home. Instead, she went to the cemetery."-Hispanic participant

"I know of a few people who have had the surgery and they've had to have it three and four times."-Hispanic participant

"I see the results friends have. I had a friend who got the shots and it didn't work at all." -Hispanic participant

"I know of my sister-in-law, who had it done [knee replacement surgery] and she's heavier than I am, and it didn't work for her. So I said I [would] rather cope with my pain before subjecting myself to surgery."-Hispanic participant.

"My sister had an injection and the doctor didn't apply it correctly. It seems he was a resident and he didn't do it correctly... I was never going to get the injection because of her experience, and I won't let them do it to me. Not even if I were crazy would I let them do it."-Hispanic participant

There was considerable discussion in all groups about who made health care decisions - the patient, the physician or others. Most of these comments (72% across all groups) supported the theme that the primary decision maker in the doctor-patient relationship is the physician.

"The doctor's recommendation is very important to me because I think that we should respect each professional in their field. He's the one with the knowledge in the field. So, if as a doctor, he recommends you can have surgery, it's because he's very sure within his professional field, about what he's recommending. He has read. He has studied, and knows what the advantages and disadvantages are..."-Hispanic participant

"The pain doctor. I've only seen him once, but I felt great confidence. He took his time with me and he made me feel like I was important to him. With a doctor like that, I think I would take the risk to get the injection or try something else because I feel confident." -Hispanic participant

"I have a lot of physicians in my family. So I listen to them fairly routinely. But I also question them quite seriously." - non-Hispanic White participant.

#### Doctor-patient relationship

There was considerable discussion in each focus group about patients' trust of these physicians and about the extent of satisfaction or dissatisfaction with the physician's advice, the physician's manner and rapport and the service provided by the physician's practice. In general, patients trusted their physicians. This theme recurred consistently across the six groups. The non-Hispanic White back pain group made many more statements expressing dissatisfaction with the physician's advice than statements expressing satisfaction with the physician's advice (Table 2).

"I don't want to be talked down to. Don't throw out a bunch of medical jargon at me...to be dismissed because you're just a patient, and I'm the doctor and I'm the god, doesn't work for me. I think I was more afraid of having him do the surgery than I was, actually, of having the surgery." - Non-Hispanic participant

"I went to see him. He stayed with me maybe all of five or eight minutes. 'Do this, do that. Bop bop bop. Can you do this? Can you do that? Bend over. Looked at my MRI. Three minutes longer and he was out of there. After four months. That was my experience." Non-Hispanic White participant

This pattern was not seen in knee pain groups or in Hispanic groups. Across all clinical and ethnic groups, there was a fairly even distribution of statements expressing satisfaction with the physician's manner and rapport as compared with dissatisfaction.

Finally, subjects were more likely to comment on their dissatisfaction than their satisfaction with the service provided by the medical practice. This pattern was roughly similar across all groups.

- "With my spine doctor, I am disillusioned because I have to wait an hour, an hour and a half to be seen. Therefore, that's a bit discouraging, and secondly, everything is in a rush."-Hispanic participant

"What makes me angry is you wait about 45 minutes for the doctor to see you for five minutes. By the time you open your mouth to say something, your time is up, and he goes to see another patient, and then another. He doesn't spend enough time with me. I want to let him know that I am in pain."-Hispanic participant

#### Coping resources

The Hispanic focus group participants were considerably more likely to discuss the ways in which they cope with their musculoskeletal problem than the non-Hispanic Whites (33 comments among Hispanics vs. 1 among non-Hispanic Whites). The issue of coping came up more frequently among Hispanics with back pain than among those with knee pain. Subjects noted a number of different coping strategies including having a positive attitude, faith and religion and having family members care for patients in the event they have surgery.

"When I went for the first shot in my back, I had the chance to see the size of the needle. I put myself in God's hands."-Hispanic participant

"Well, we have to count on Him up there. We have to rely on the one up there first, and then, on the doctors."-Hispanic participant

"Surgery? It would be in the name of the Lord because many people tell me not to have surgery because I can remain handicap[ped], or something."-Hispanic participant

#### Information sources

Subjects provided a range of comments about the types of media they prefer for receiving health information. By far the most preferred source of information, across all focus groups, was the physician. Print media were less frequently preferred and DVD even less frequently. Preference for internet-based approaches differed across focus groups. Non-Hispanic whites and the less vulnerable Hispanics commented that the internet was an important source of health information.

"You know with the Internet everybody just goes online...Before you even get to the doctor you can do a lot of research ahead of time."- Non-Hispanic White participant

"I would be very likely to [go to the website], and I would read a pamphlet. If it presented both sides, it would be very useful, if it presented alternatives."-Non-Hispanic White participant

On the other hand, Hispanics with vulnerability sores of two or greater reported much lower use of the Internet.

"I'm kind of old-fashioned and I don't [use the Internet]."-Hispanic participant"

## Discussion

We conducted focus groups among Hispanics and non-Hispanic Whites with chronic back and knee pain. We chose these conditions because management decisions are typically preference-based and because patients with these conditions must discuss a range of potentially risky decisions with their providers including corticosteroid injections and surgical procedures. We analyzed the focus group data using a content analysis approach, permitting the data to drive hypothesis generation.

The research highlighted several important differences between Hispanics and non-Hispanic Whites in regard to health-related decision-making. Hispanics were more likely to be influenced by word-of-mouth communication of bad experiences with certain treatments, making them disinclined to seek or choose those treatments. Hispanics were more likely than Whites to comment on strategies for coping with musculoskeletal problems including a positive outlook, faith, religion, and family support. Finally, the internet served as a source of health information for Whites and Hispanics with low vulnerability scores but not for Hispanics with high vulnerability scores.

Our findings also point to important commonalities. The data suggest that pain and functional limitation are the central experiences of these musculoskeletal disorders, shared across ethnic groups. In addition, across all strata subjects indicated that, in general, they trust their physicians. Across all strata, subjects provided mixed reviews of their physicians' manner and rapport and they provided largely negative assessments of office practices.

These findings are largely consistent with prior literature. In a population-based survey, Levinson and colleagues noted that Hispanics are more likely than non-Hispanic Whites to prefer that their physicians make health-related decisions [19]. Similarly, Xu et al noted that Hispanics were less likely to report a participatory relationship with their physicians than non-Hispanic Whites [25]. Our data demonstrate that across all ethnic groups in this study, about three quarters of patients see themselves as primary decision-makers. In a study of decision-making surrounding total knee replacement, Kroll and colleagues found that Hispanic patients developed trust in the physician in part based on word-of-mouth testimonials of friends and family. This echoes our finding of the importance of word of mouth in the Latino community [17].

Our findings can be viewed from the perspective of the Ottawa Decision Support Framework, which emphasizes patients' needs for knowledge, resolution of decisional conflict, involvement in the decision process and sources of support for their decisions as well as the importance of value consonant decisions [15,16]. We identified differences in strategies for seeking knowledge, with Hispanics more likely to use word of mouth and less likely to use the internet. Differences surfaced as well in sources of support with Hispanics more likely to seek support from family and religion.

These data have implications for efforts to improve preference-based decision-making. Patients across all groups view their physicians as trusted sources, highlighting the important role of physicians in efforts to promote optimal decision-making. Word of mouth is a powerful source of information among Hispanics; decision support should target this potential source of misinformation. Faith, religion and family appear to be especially important among Hispanics, suggesting that these sources of support could be integrated into decision support among Hispanics. Finally, Hispanics who are likely less acculturated (those with two or more vulnerability factors) are much less likely to respond to internet-based strategies for providing health information.

Our study has several noteworthy limitations. Our budget did not permit multiple focus groups within each combination of condition and ethnicity. While we observed saturation of comments, we acknowledge that more focus groups might have provided additional insights. We note that the neighborhood-level socioeconomic information that we used for socioeconomic stratification may not reflect individual characteristics accurately. In fact, 86% of non-Hispanic Whites had attended college as compared with 24% of Hispanics. These imbalances require that differences we observed be attributed cautiously to Hispanic ethnicity. The inclusion of two clinical conditions (back and knee pain) may improve generalizability but also introduces an element of heterogeneity to the analysis with respect to clinical features and age that makes interpretation less incisive. Finally, we recognize that the Hispanic community in the U.S. is highly heterogeneous. Our Boston-based sample of Hispanics (comprised largely of patients of Dominican and Puerto Rican descent) cannot account for the variability in the U.S. Hispanic population with respect to nation of origin, duration of residence in the U.S., facility with English, acculturation or educational attainment. For all these reasons, the findings should be confirmed in further research and generalized cautiously.

## Conclusion

In conclusion, patients with musculoskeletal problems share a core of concerns revolving around the primacy of pain and pain relief. However, Hispanic patients with knee and back pain appear to differ from non-Hispanic Whites in areas pertinent to shared decision-making, including the role of adverse experiences in shaping treatment preferences, the importance of family and religion in the decision process, and preferences for use of internet sources of health information as the foundation for health decisions. These findings should be confirmed, as they may help to shape strategies for refining the health-related decision-making process among Hispanics.

## Competing interests

The authors declare that they have no competing interests.

## Authors' contributions

JNK wrote the grant and paper and led all aspects of the research. NL and JS led in the development of the moderator's guide, assisted in leading the focus groups and edited the manuscript. LSW moderated the non-Hispanic focus groups, assisted in development of the moderator's guide, performed the fist level analysis of transcripts, drafted many paragraphs of the manuscript and edited all of it. PE recruited patients into the study, assisted in drafting the moderator's guide and edited the mansucript critically. HLH and KLC performed the quantitative coding of the transcript data and edited the final manuscript critically. AE provided expertise on Hispanic health and performing research studies involving Hispanics and also edited the final manuscript critically. EL oversaw all methodologic aspects of the study including the development of vulnerability factors using zip code data and also edited the final manuscript critically.

All authors read and approved the final manuscript.

## Pre-publication history

The pre-publication history for this paper can be accessed here:

http://www.biomedcentral.com/1471-2474/12/78/prepub
